# Laboratory as Aid in Contagious Disease Admissions in Cantonment Hospitals

**Published:** 1919

**Authors:** 


***Laboratory as Aid in Contagious Disease Admissions in Cantonment Hospitals.*** By Major A. B. MORILL. *Journal of the American Medical Association*, Sept. 28, 1918.

The author speaks of the difficulty of grouping the types of contagious cases and of the resulting errors of diagnosis. At Camp Grant, it was suggested to make the white blood count for the differentiation between diseases at the receiving office so that patients should be sent directly to the proper ward. A unit laboratory was placed in the receiving office and a leukocyte count was made in all doubtful cases, subsequently in all cases.

*Work of the Laboratory.* Cultures and cover glass smears are made from all sore throats to determine the presence of the diphtheria bacilli. In all cases of sore throat with exudates showing organisms resembling the diphtheria bacilli, the patients are immediately given antitoxin. Blood is obtained for routine Wassermann tests which has recently proved of value for the determination of the frequency of the hemolytic streptococcus. A table is given showing the average white counts in the various diseases on admission, and grouping the cases into complicated and uncomplicated cases.

The objection that laboratory tests undesirably lengthen the process of getting the patients to bed can hardly be put forward, for should it be the case, it could easily be remedied by increasing the laboratory staff. Installation of small cubicles will maintain the technic of the masking system, while the patient is unmasked for throat cultures, etc. These cubicles should be made of wood and sheet metal and covered with white enamel paint, so that they can be frequently disinfected.

